# Association between anemia and 1-year recurrence of paroxysmal atrial fibrillation after radiofrequency ablation

**DOI:** 10.1515/jtim-2025-0042

**Published:** 2025-10-16

**Authors:** Enze Li, Zhen Cao, Dongze Li, Zixu Zhao, Chao Jiang, Xiaoxia Liu, Caihua Sang, Changsheng Ma

**Affiliations:** Department of Cardiology, Beijing Anzhen Hospital, Capital Medical University, National Clinical Research Center for Cardiovascular Diseases, Beijing, China; Department of Cardiology, The First Affiliated Hospital, Harbin Medical University, Harbin, Heilongjiang Province, China

## Introduction

Atrial fibrillation (AF) is the most common tachyarrhythmia, and it imposes a significant burden on the healthcare system. Previous randomized clinical trials have shown that early radiofrequency catheter ablation (RFCA) alleviates AF-related symptoms and reduces mortality and cerebral infarction rates.^[1]^ However, AF recurrence risk remains high even after RFCA.

Anemia is prevalent among AF patients with approximately 16% affected. At the molecular level, systemic iron in mammals is predominantly transported by transferrin, while cellular iron uptake depends on the interaction between transferrin and transferrin receptor protein 1. Transferrin receptor protein 1 gene knockout-induced anemia triggers lethal cardiomyopathy due to cardiomyocyte metabolic dysfunction, characterized by impaired oxidative phosphorylation and defective mitophagy.^[2]^ Prior studies have also shown that anemia increases all-cause and cardiovascular mortality in AF patients, and reduces post-ablation sinus rhythm maintenance rates. However, the recurrence risk following RFCA in patients with concurrent anemia and paroxysmal atrial fibrillation (PAF) remains uninvestigated.^[3]^

Here, we evaluated the impact of anemia on AF recurrence after RFCA, and further explored how gender, age and other factors influence the association between anemia and post-ablation recurrence in AF patients.

## Materials and methods

The Chinese-AF Registry (NCT06987825) is a multicenter registry enrolling consecutive AF patients from 32 hospitals in Beijing including 18 centers offering ablation.^[4]^ This study included 3078 PAF patients undergoing first-time RFCA (2018–2021), excluding those < 18 years, less than 6-month follow-up, persistent AF, valvular heart disease, or missing Hb data. Anemia was defined according to World Health Organization (WHO)’s criteria: Hb < 130 g/L (men) or < 120 g/L (women).

Baseline data were collected *via* electronic case report forms. Comorbidities including hypertension (HTN), diabetes, and chronic heart failure (CHF). Hb and eGFR were recorded, with anemia status determined from pre-procedure blood tests within 1 year.

RFCA was perfor med using a 3D electroanatomic mapping system (CARTO3), with pulmonary vein isolation as the core procedure. Additional ablation was tailored to individual cases. Anticoagulation was administered for ≥3 months, and NOACs were recommended for CHA_2_DS_2_-VASc ≥2. Antiarrhythmic agents were used during the blanking period and discontinued at 3 months.

Follow-up included daily ECG for 48 hours post-procedure, monthly 24-hour Holter monitoring for 3 months, and biannual ECG/Holter for up to 1 year. AF recurrence was defined by Chinese-AF Registry as ≥ 30-second atrial arrhythmia on ECG.

Statistical analyses used Wilcoxon rank sum tests for continuous variables while Pearson’s Chi-squared/Fisher’s exact tests for categoricals. Subgroup analyses stratified by age, sex, HTN, and BMI were performed. Cox regression models (univariate/multivariate) assessed HRs for AF recurrence, with interaction tests and KM curves. Adjusted models included gender, age, BMI, and comorbidities. Analyses were conducted using SPSS 22.0 (IBM), with *P* < 0.05 considered significant.

## Results

### Baseline characteristics

A total of 3078 PAF patients in the Chinese-AF cohort undergoing de novo RFCA met our eligibility criteria. The average follow-up time was 313.16 ± 93.60 days, and 123 (4.0%) of the patients were diagnosed with anemia after initial RFCA. Study population characteristics, stratified by anemia, are summarized in Supplementary Table S1.

Patients with anemia were significantly older [65.70 (56.7071.70) *vs*. 62.60 (55.30-68.00) years; *P* < 0.001], more likely to be female (82.1% *vs*. 36.6%; *P* < 0.001), and had lower BMI [24.19 (22.21–26.04) *vs*. 25.27 (23.42–27.44) kg/m^2^; *P* = 0.002] compared to non-anemic patients.

### AF recurrence

**Figure 1:**
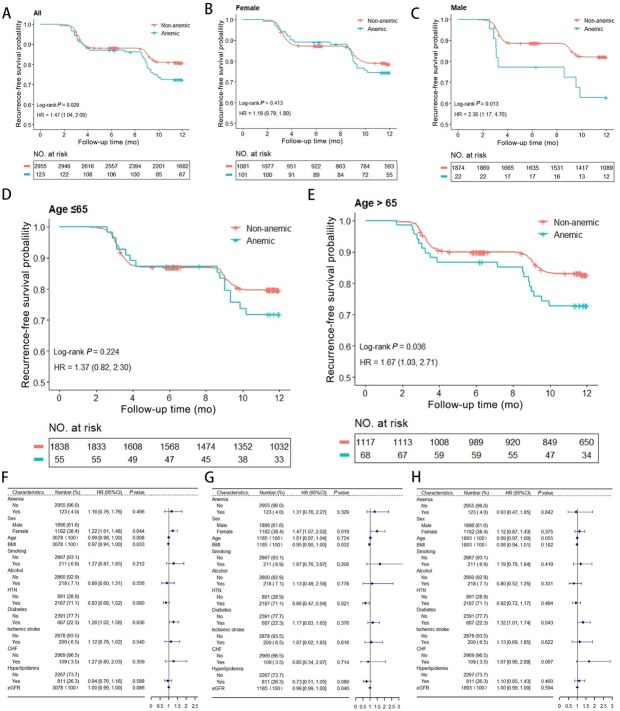
AF Recurrence Risk Analysis. (A) Kaplan-Meier survival analysis of AF recurrence of all groups. (B-C) Subgroup analyses stratified by sex. (D-E) Subgroup analyses stratified by age. (F) Analysis of anemia as an independent risk factor for AF recurrence. (G-H) Age-stratified analysis showing anemia’s association with recurrence (left for ≤ 65, right for > 65). AF: Atrial fibrillation; HR: Hazard ratio; CI: Confidence interval.

33 patients (26.8%) in the anemia group and 551 patients (18.6%) in the non-anemic group experienced AF recurrence following initial RFCA during a total of 2640 person-years of follow-up. The Kaplan-Meier (KM) survival analysis and a Cox regression model for AF recurrence is shown in Figure 1A, and indicates a higher risk of recurrence in anemic patients, with a HR of 1.47 (log-rank, *P* = 0.029; 95%CI: 1.04–2.09). Subgroup analyses stratified by gender (Figure 1B and 1C) and age (Figure 1D and 1E) revealed that this relationship was only present in male patients (log-rank, *P* = 0.013; HR: 2.36, 95%CI: 1.17–4.76) and those aged ＞ 65 years (log-rank, *P* = 0.036; HR: 1.67, 95%CI: 1.03–2.71). In contrast, the association was not significant in female patients (log-rank, *P* = 0.413; HR: 1.19, 95%CI: 0.79–1.80) or those aged ≤ 65 years (log-rank, *P* = 0.224; HR: 1.37, 95%CI: 0.82–2.30).

Anemia was not an independent risk factor for AF recurrence (HR: 1.16, 95%CI: 0.76–1.76, *P* = 0.496) (Figure 1F). Notably, factors such as younger age (HR: 0.99, 95%CI: 0.98–0.99, *P* = 0.009), indicating a 1% decrease in recurrence risk per year of age. Plus, lower BMI (HR: 0.97, 95%CI: 0.94–0.99, *P* = 0.033), and diabetes (HR: 1.26, 95%CI: 1.02–1.56, *P* = 0.036) were significantly associated with recurrence of AF.

### Subgroup analyses

Interaction analysis revealed that anemia may increase AF recurrence risk in males (HR: 2.36, 95%CI: 1.17–4.75, *P* = 0.017), those over 65 years (HR: 1.67, 95%CI: 1.03–2.71, *P* = 0.038), those with HTN (HR: 1.72, 95%CI: 1.15–2.58, *P* = 0.008), and those with BMIs < 25 kg/m^2^(HR: 1.72, 95%CI: 1.08–2.74, *P* = 0.023). However, anemia was not found to increase AF recurrence risk in other groups (Table 1).

**Table 1:** Baseline characteristics and interactions in the relationship between anemia and AF recurrence risk

Variable/subgroup	Anemia (*N* = 123) (Baseline)	Non-Anemia (*N* = 2955) (Baseline)	Overall (*N* = 3078) (Baseline)	*P*-value (Baseline Difference)	Subgroup *n* (%)	AF recurrence in non- anemia (events/ total)	AF recurrence in anemia (events/ total)	HR (95%CI) for anemia (AF recurrence)	*P* (for HR)	*P* for interaction
All patients					3078 (100.00)	551/2955	33/123	1.47 (1.04–2.09)	0.031	
Age (years)	65.70 (56.70–71.70)	62.60 (55.30–68.00)	62.60 (55.40–68.20)	< 0.001^b^						0.596
> 65					1185 (38.50)	186/1117	18/68	1.67 (1.03–2.71)	0.038	
< 65					1893 (61.50)	365/1838	15/55	1.38 (0.82–2.30)	0.227	
Female	101 (82.11%)	1081 (36.58%)	1182 (38.40%)	< 0.001^a^						0.099
Male					1896 (61.60)	327/1874	8/22	2.36 (1.17–4.75)	0.017	
Female					1182 (38.40)	224/1081	25/101	1.19 (0.79–1.80)	0.414	
BMI (kg/m^2^)	24.19 (22.21–26.04)	25.27 (23.42–27.44)	25.25 (23.38–27.41)	0.002^b^						0.143
> 30					215 (8.28)	36/208	1/7	0.76 (0.10–5.51)	0.782	
< 25					1210 (46.57)	229/1151	19/59	1.72 (1.08–2.74)	0.023	
25–30					1173 (45.15)	208/1141	4/32	0.66 (0.25–1.78)	0.411	
Medical history										
Ischemic stroke	8 (6.50%)	192 (6.5%)	200 (6.50%)	1.000^a^						0.430
No					2878 (93.50)	514/2763	30/115	1.42 (0.98–2.05)	0.062	
Yes					200 (6.50)	37/192	3/8	2.39 (0.74–7.76)	0.147	
CHF	7 (5.69%)	102 (3.45%)	109 (3.50%)	0.205^c^						0.333
No					2969 (96.46)	529/2853	30/116	1.41 (0.98–2.04)	0.068	
Yes					109 (3.54)	22/102	3/7	2.63 (0.78–8.79)	0.117	
HTN	87 (70.73%)	2100 (71.07%)	2187 (71.10%)	0.936^a^						0.193
No					891 (28.95)	186/855	8/36	1.00 (0.49–2.04)	0.993	
Yes					2187 (71.05)	365/2100	25/87	1.72 (1.15–2.58)	0.008	
CAD	22 (17.89%)	357 (12.08%)	379 (12.30%)	0.055^a^						
Hyperlipidemia	37 (30.08%)	774 (26.19%)	811 (26.3%)	0.338^a^						0.772
No					2267 (73.65)	414/2181	24/86	1.53 (1.01–2.31)	0.043	
Yes					811 (26.35)	137/774	9/37	1.36 (0.69–2.67)	0.372	
Diabetes	33 (26.83%)	654 (22.13%)	687 (22.3%)	0.220^a^						0.900
No					2391 (77.68)	415/2301	23/90	1.44 (0.95–2.19)	0.087	
Yes					687 (22.32)	136/654	10/33	1.51 (0.80–2.88)	0.206	
Vital sign and laboratory tests										
Heart rate (bpm)	70.00 (61.00–78.00)	72.00 (64.00–80.00)	70.00 (61.00–78.00)	0.024^b^						
eGFR [mL/(min 1.73 m^2^)]	106.42 (86.36–130.93)	112.68 (98.1–128.77)	112.60 (97.88–128.83)	0.058^b^						0.293
> 90					2600 (84.69)	446/2513	24/87	1.61 (1.07–2.43)	0.023	
< 60					35 (1.14)	8/24	3/11	0.77 (0.21–2.92)	0.705	
60-90					435 (14.17)	93/411	5/24	0.87 (0.35–2.13)	0.753	
Hb (g/L)	114.00 (109.00–117.00)	147.00 (137.00–157.00)	146.00 (136.00–157.00)	<0.001^b^						
Adverse personal habits										
Smoking	1 (0.81%)	210 (7.11%)	211 (6.90%)	0.007^a^						0.988
No					2867(93.14)	506/2745	33/122	1.51 (1.06–2.14)	0.023	
Yes					211 (6.86)	45/210	0/1	0.00 (0.00–Inf)	0.997	
Alcohol use	1 (0.81%)	217 (7.34%)	218 (7.10%)	0.006^a^						0.987
No					2860 (92.92)	511/2738	33/122	1.48 (1.04–2.10)	0.029	
Yes					218 (7.08)	40/217	0/1	0.00 (0.00–Inf)	0.996	
Other anticoagulants and antithrombotic drugs										
NOAC	78 (63.41%)	1724 (58.34%)	1802 (58.5%)	0.263^a^						
Warfarin	4 (3.25%)	8 (0.27%)	12 (0.40%)	< 0.001^c^						
Aspirin	1 (0.81%)	8 (0.27%)	9 (0.30%)	0.308^c^						
Clopidogrel	10 (8.13%)	144 (4.87%)	154 (5.00%)	0.104^a^						

^a^Pearson's Chi-squared test; ^b^Wilcoxon rank sum test; ^c^Fisher's exact test. Continuous variables are presented as median with IQR. Categorical variables are expressed as numbers and proportions; *P*-value (baseline difference) refers to between-group differences for anemia vs. non-anemia based on Wilcoxon rank sum tests, Fisher's exact tests or Pearson's Chi-squared tests; HRs (95% CIs) for anemia (AF recurrence) were estimated using Cox proportional hazard model without adjustment for any covariate; *P* (for HR) refers to the *P*-value for the hazard ratio; *P* for Interaction tests whether the association between anemia and AF recurrence risk differs across subgroups of the variable; AF: Atrial fibrillation; IQR: interquartile range; BMI: body mass index; CHF: chronic heart failure; HTN: hypertension; CAD: coronary artery disease; eGFR: estimated glomerular filtration rate; Hb: hemoglobin; NOAC: non-vitamin K antagonist oral anticoagulant; HR: hazard ratio; CI: confidence interval; For smoking (Yes) and alcohol use (Yes) subgroups with anemia, the event count was 0/1, leading to an HR of 0.00 and a *P*-value of 0.997/0.996 respectively; the CI (0.00 – Inf) reflects this; Baseline *P*-values in bold from original Table 1 (e.g. age < 0.001, female < 0.001, BMI 0.002, heart rate 0.024, Hb < 0.001, smoking 0.007, alcohol use 0.006, warfarin < 0.001) are not specially bolded here due to plain text limitations but were significant.

Additionally, age stratification indicated that anemia had a moderate but non-significant association with recurrence in patients aged > 65 years (HR: 1.31, 95%CI: 0.76–2.27, *P* = 0.330) and it was not significantly associated with recurrence in patients aged ≤ 65 years (HR: 0.93, 95%CI: 0.47–1.85, *P* = 0.842) (Figure 1G-1H).

## Discussion

Anemia is well-recognized as an independent risk factor for adverse cardiovascular outcomes in heart failure, coronary artery disease, and atherosclerosis. It frequently occurs in AF patients, especially those on oral anticoagulants. Previous research linked anemia to higher AF recurrence risk, showing independent association during 23 months of follow-up.^[5]^ Arrhythmogenic mechanisms vary by anemia types. Iron-deficiency anemia promotes AF through tissue hypoxia-induced atrial remodeling and electrophysiological instability, as supported by recent studies showing increased atrial fibrosis on cardiac MRI in anemic patients^[6]^ and higher arrhythmia burden on Holter monitoring.^[7]^ Thalassemia may act *via* iron overload or oxidative stress^[8]^, though its direct causal role remains unclear. Hemodilution resulting from intravenous fluid administration elevates systemic levels of inflammatory factors, hypoxia, and oxidative stress markers. It may also potentially induce AF recurrence.^[9]^

To our knowledge, this is the first study identifying sex-associated effects of anemia on post-ablation PAF recurrence. Subgroup analysis revealed a anemia-recurrence association in men (*P* < 0.01) but not in women overall. However, among women (> 65 years), anemia correlated with higher recurrence risk (*P* < 0.05). Women often have a higher prevalence of anemia, which can be attributed to factors such as menstrual blood loss, pregnancy demands, or differences in iron intake. Hormonal differences, particularly the decline in estrogen after menopause, may influence AF susceptibility and could interact with conditions like anemia. While estrogen has been suggested to have protective cardiovascular effects, its specific role in mitigating anemia’s impact on AF recurrence in this context requires further elucidation. Men may exhibit electrophysiological characteristics, such as potentially shorter atrial refractory periods and faster conduction velocities, that could increase susceptibility to arrhythmogenic triggers like hypoxia-induced electrical instability.^[10]^ Sex-specific patterns of atrial remodeling, including potential differences in the development of hypertrophy and fibrosis, particularly in the context of stressors like anemic conditions, may amplify arrhythmogenic risk in men. In elderly women, hemodynamic factors could promote atrial remodeling and left atrial dysfunction, exacerbating AF progression,^[11]^ though sex-specific modifiers require further clarification.

Disparities in comorbidities between sexes may also ramify differences in prevalence of conditions like hypertension, coronary artery disease (CAD), and diabetes are often observed and could synergize with anemia to influence atrial remodeling. Common cardiovascular comorbidities can further compound the effects of anemia. For example, hypertension increases myocardial stress and coronary artery disease can worsen ischemia, both of which may interact with anemia-related hypoxia to adversely affect the atrial substrate. This likely contribute to the observed male-specific anemia-recurrence association. KM and subgroup data highlight anemia as an independent predictor of AF recurrence in men and elderly women. Preoperative anemia assessment, particularly iron status evaluation, is therefore critical for these populations. Correcting anemia through iron supplementation may optimize RFCA outcomes by reducing remodeling and arrhythmogenic potential. Notably, the China-AF Registry primarily includes East Asian patients, and the observed association between anemia and AF recurrence may have population-specific characteristics. While racial differences in genetic predisposition, comorbidity profiles, or treatment patterns may influence the generalizability of our findings. Future research in diverse populations is needed to validate these results and explore underlying mechanisms.

In conclusion, our study has demonstrated that patients with baseline anemia had a higher risk of post-RFCA AF recurrence compared to patients without anemia. Moreover, our subgroup analysis identified male gender, age over 65 years, hypertension, and lower BMI as significant factors that amplify anemia’s association with AF recurrence. Based on our findings, we recommend risk stratification and age- and sex-specific management strategies for AF patients undergoing ablation. Particular attention should be given to addressing anemia in men and elderly women to reduce the recurrence risk of AF.

## Supplementary Information

Supplementary materials are only available at the official site of the journal (www.intern-med.com).

## Supplementary Material

Supplementary Material Details

## References

[1] Ma C, Wu S, Liu S, Han Y (2024). Chinese guidelines for the diagnosis and management of atrial fibrillation. Pacing Clin Electrophysiol.

[2] Li J, Wang K, Starodubtseva MN, Nadyrov E, Kapron CM, Hoh J (2021). Complement factor H in molecular regulation of angiogenesis. Med Rev.

[3] Zhang Z, Jiang C, He L, Bai Y, Wu J, Hu R (2022). Associations of anemia with death and major bleeding in patients with atrial fibrillation: A report from the Chinese Atrial Fibrillation Registry Study. Clin Cardiol.

[4] Du X, Ma C, Wu J, Li S, Ning M, Tang R (2016). Rationale and design of the Chinese Atrial Fibrillation Registry Study. BMC Cardiovasc Disord.

[5] Kim M, Hong M, Kim JY, Kim IS, Yu HT, Kim TH (2020). Clinical relationship between anemia and atrial fibrillation recurrence after catheter ablation without genetic background. Int J Cardiol Heart Vasc.

[6] Hanna-Rivero N, Tu SJ, Elliott AD, Pitman BM, Gallagher C, Lau DH (2022). Anemia and iron deficiency in patients with atrial fibrillation. BMC Cardiovasc Disord.

[7] Tuncer M, Gunes Y, Guntekin U, Gumrukcuoglu HA, Eryonucu B, Guler N (2009). Heart rate variability in patients with iron deficiency anemia. Arq Bras Cardiol.

[8] Tu SJ, Hanna-Rivero N, Elliott AD, Clarke N, Huang S, Pitman BM (2021). Associations of anemia with stroke, bleeding, and mortality in atrial fibrillation: A systematic review and meta-analysis. J Cardiovasc Electrophysiol.

[9] Cakir MU, Yavuz-Aksu B, Aksu U (2023). Hypervolemia suppresses dilutional anaemic injury in a rat model of haemodilution. J Transl Int Med.

[10] Gillis AM (2017). Atrial Fibrillation and Ventricular Arrhythmias: Sex Differences in Electrophysiology, Epidemiology, Clinical Presentation, and Clinical Outcomes. Circulation.

[11] Ko D, Rahman F, Schnabel RB, Yin X, Benjamin EJ, Christophersen IE (2016). Atrial fibrillation in women: epidemiology, pathophysiology, presentation, and prognosis. Nature Reviews Cardiology.

