# Supplementary material for: Association between anemia and 1-year recurrence of paroxysmal atrial fibrillation after radiofrequency ablation
Source: J Transl Int Med. 2025 Oct 16;13(5):480–5. doi: 10.1515/jtim-2025-0042 (PMC12569575; doi:10.1515/jtim-2025-0042)
Supplement: Supplementary file 1 — Supplementary Material Details [file jtim-2025-0042_sm.pdf]

Association between anemia and 1-year recurrence of paroxysmal atrial fibrillation after radiofrequency ablation

Enze Li[1], Zhen Cao[1], Dongze Li[2], Zixu Zhao[1], Chao Jiang[1], Xiaoxia Liu[1], Caihua Sang[1], Changsheng Ma[1]

[1]Department of Cardiology, Beijing Anzhen Hospital, Capital Medical University, National Clinical Research Center for Cardiovascular Diseases, Beijing 100029, China

[2]Department of Cardiology, The First Affiliated Hospital, Harbin Medical University, Harbin 150001, Heilongjiang Province, China

Address for Correspondence: Changsheng Ma, Department of Cardiology, Beijing Anzhen Hospital, Capital Medical University, No.2 Anzhen Rd, Chaoyang District, Beijing 100029, China. E-mail: chshma@vip.sina.com

Supplementary Material

Table S1: Patient baseline characteristics.

Baseline characteristic	Total number			*P*-value
	**Anemia (*N*=123)**	**Non-Anemia (*N*=2955)**	**Overall (*N*=3078)**	
Age (years)	65.70 (56.70 ~ 71.70)	62.60 (55.30 ~ 68.00)	62.60 (55.40 ~ 68.20)	**<0.001[2]**
Female	101 (82.11%)	1081(36.58%)	1182(38.40%)	**<0.001[1]**
BMI (kg/m^2)	24.19 (22.21 ~ 26.04)	25.27 (23.42 ~ 27.44)	25.25 (23.38 ~ 27.41)	**0.002[2]**
Medical history				
Ischemic stroke	8(6.50%)	192(6.5%)	200 (6.50%)	**1.000[1]**
CHF	7(5.69%)	102(3.45%)	109 (3.50%)	**0.205[3]**
HTN	87(70.73%)	2100(71.07%)	2187 (71.10%)	**0.936[1]**
CAD	22(17.89%)	357(12.08%)	379 (12.30%)	**0.055[1]**
Hyperlipidemia	37(30.08%)	774(26.19%)	811 (26.3%)	**0.338[1]**

Diabetes	33(26.83%)	654(22.13%)	687 (22.3%)	**0.220**[1]
Vital sign and laboratory tests				
Heart rate (bpm)	70.00 (61.00 ~ 78.00)	72.00 (64.00 ~ 80.00)	70.00 (61.00 ~ 78.00)	**0.024**[2]
eGFR [ml/(min 1.73m^2)]	106.42 (86.36 ~ 130.93)	112.68 (98.19 ~ 128.77)	112.60 (97.88 ~ 128.83)	**0.058**[2]
Hb (g/L)	114.00 (109.00 ~ 117.00)	147.00 (137.00 ~ 157.00)	146.00 (136.00 ~ 157.00)	**<0.001**[2]
Adverse personal habits				
Smoking	1(0.81%)	210(7.11%)	211 (6.90%)	**0.007**[1]
Alcohol use	1(0.81%)	217(7.34%)	218 (7.10%)	**0.006**[1]
Other anticoagulants and antithrombotic drugs				
NOAC	78(63.41%)	1724(58.34%)	1802 (58.5%)	**0.263**[1]
Warfarin	4(3.25%)	8(0.27%)	12 (0.40%)	**<0.001**[3]
Aspirin	1(0.81%)	8(0.27%)	9 (0.30%)	**0.308**[3]
Clopidogrel	10(8.13%)	144(4.87%)	154 (5.00%)	**0.104**[1]

[1]Pearson's Chi-squared test

[2]Wilcoxon rank sum test

[3]Fisher's exact test

Continuous variables are presented as median with IQR. Categorical variables are expressed as numbers and proportions. Between-group differences, based on Wilcoxon rank sum tests, Fisher's exact tests or Pearson's Chi-squared tests, are labeled in bold. IQR: interquartile range; BMI: body mass index; CHF: chronic heart failure; HTN: hypertension; CAD: coronary artery disease; eGFR: estimated glomerular filtration rate; Hb: hemoglobin; NOAC: non-vitamin K antagonist oral anticoagulant.
